# Cannabinoids as Glial Cell Modulators in Ischemic Stroke: Implications for Neuroprotection

**DOI:** 10.3389/fphar.2022.888222

**Published:** 2022-06-01

**Authors:** Andrés Vicente-Acosta, Maria Ceprian, Pilar Sobrino, Maria Ruth Pazos, Frida Loría

**Affiliations:** ^1^ Centro de Biología Molecular Severo Ochoa (CSIC-UAM), Madrid, Spain; ^2^ Departamento de Biología Molecular, Universidad Autónoma de Madrid, Madrid, Spain; ^3^ ERC Team, PGNM, INSERM U1315, CNRS UMR5261, University of Lyon 1, University of Lyon, Lyon, France; ^4^ Departamento de Neurología, Hospital Universitario Fundación Alcorcón, Alcorcón, Spain; ^5^ Laboratorio de Apoyo a la Investigación, Hospital Universitario Fundación Alcorcón, Alcorcón, Spain

**Keywords:** cannabinoids, neuroinflammation, ischemic stroke, glia, drug target

## Abstract

Stroke is the second leading cause of death worldwide following coronary heart disease. Despite significant efforts to find effective treatments to reduce neurological damage, many patients suffer from sequelae that impair their quality of life. For this reason, the search for new therapeutic options for the treatment of these patients is a priority. Glial cells, including microglia, astrocytes and oligodendrocytes, participate in crucial processes that allow the correct functioning of the neural tissue, being actively involved in the pathophysiological mechanisms of ischemic stroke. Although the exact mechanisms by which glial cells contribute in the pathophysiological context of stroke are not yet completely understood, they have emerged as potentially therapeutic targets to improve brain recovery. The endocannabinoid system has interesting immunomodulatory and protective effects in glial cells, and the pharmacological modulation of this signaling pathway has revealed potential neuroprotective effects in different neurological diseases. Therefore, here we recapitulate current findings on the potential promising contribution of the endocannabinoid system pharmacological manipulation in glial cells for the treatment of ischemic stroke.

## Introduction

Stroke is a rapidly developing neurological pathology that involves the appearance of clinical symptoms due to a global or focal disturbance of brain function, generally with vascular origin (Sacco et al., 2013). It is the second leading cause of death worldwide after coronary and heart disease, constituting 10% of total mortality, the first cause of disability, and the second cause of dementia, causing a significant family, healthcare, and socioeconomic cost (Feigin, 2021). Moreover, data from different studies indicate that stroke prevalence is increasing and will continue to rise, probably due to an increment in life expectancy globally (Feigin, 2021). Stroke is a heterogeneous disease and depending on the nature of the brain injury, two major types can be distinguished, hemorrhagic stroke (15% of cases) and ischemic stroke (85% of cases), which in some cases could be transient ischemic attacks (van Asch et al., 2010; Meschia and Brott, 2018). Hemorrhagic stroke is caused by the rupture of a blood vessel in the brain and ischemic stroke, the focus of this review, occurs when a cerebral artery is occluded by a blood clot, causing a cerebral infarction (Pekny et al., 2019). Thrombus formation can have different etiologies, such as atherothrombosis, cardioembolism, small vessel occlusive disease, rare cause (infection, neoplasia, myeloproliferative syndrome, metabolic disorders, coagulation disorders) or even cryptogenic stroke (Díez Tejedor et al., 2001; Meschia and Brott, 2018). As the brain and its proper functioning depend on an adequate supply of oxygen and glucose, its cessation causes neuronal death and glial activation that lead to functional alterations not only in the affected area but also in related brain areas (Pekny et al., 2019).

Due to the high prevalence of ischemic stroke and to a higher opportunity for an effective therapeutic intervention than for hemorrhagic, a great effort has generally been devoted to the study of ischemic stroke. It has been observed that primary prevention, which acts on modifiable or potentially modifiable vascular risk factors, can considerably reduce the incidence of ischemic strokes. Among the modifiable risk factors, the most important are arterial hypertension, tobacco use, diabetes mellitus, hypercholesterolemia, obesity, physical inactivity, and atrial fibrillation (Kuriakose and Xiao, 2020). However, due in part to the estimated global increase in the prevalence of ischemic stroke and the need to find appropriate treatments to reduce sequelae, it is essential to thoroughly study the molecular mechanisms underlying the disease in order to find new treatments that will eventually lead to functional recovery of patients.

### Molecular Mechanisms of Ischemic Stroke

After the ischemic event, there is a central zone of irreversibly damaged tissue known as core that receives less than 15% of normal cerebral blood flow (CBF), a surrounding area of damaged tissue that may recover its function known as penumbra, receiving less than 40% of normal CBF, and the peri-infarct region, which receives between 40 and 100% of normal CBF (Meschia and Brott, 2018; Feske, 2021). In the acute phase of cerebral ischemia, precisely due to the reduced CBF, the affected tissue experiences cellular energy depletion, with a dysfunction of the ATP-dependent ionic pumps producing the intracellular accumulation of calcium (Ca^2+^). This in turn induce the release and accumulation of excitotoxic amino acids like glutamate in the extracellular space (Qureshi et al., 2003; Lai et al., 2014). As a consequence of the intracellular Ca^2+^ increase, Ca^2+^-dependent enzymes are activated, resulting in mitochondrial dysfunction and cell death, mainly by necrosis (Fernández-Ruiz et al., 2015; Belov Kirdajova et al., 2020; Kuriakose and Xiao, 2020). In this first stage, microglial cells act as early players and their activation leads to the production of pro-inflammatory mediators, such as tumour necrosis factor-alpha (TNF-α), interleukin-1beta (IL-1β), and reactive oxygen species (ROS) (Lambertsen et al., 2005; Clausen et al., 2008; Allen and Bayraktutan, 2009; Chen et al., 2019). These factors recruit other inflammatory cell populations into the ischemic area, mainly circulating monocytes, which interact with astrocytes through the secretion of cytokines and chemokines, possibly contributing to astrocyte activation (Kim and Cho, 2016; Frik et al., 2018; Hersh and Yang, 2018; Rizzo et al., 2019). Once astrocytes are activated, they shift their morphology and function according to the biological context. Indeed, increasing evidence sustains the critical role of these cells in the brain’s response to stroke (Pekny et al., 2016), but their harmful or beneficial contribution to the ischemic pathway is currently under intense debate (Liddelow et al., 2017). Astrocytes are also critical for glial scar formation surrounding the infarct zone, which may help limit immune cell infiltration (Liddelow and Barres, 2015, 2017). Moreover, vasogenic edema, characterized by extravasation and extracellular accumulation of fluid into the cerebral parenchyma caused by disruption of the blood-brain barrier (BBB), takes place during the subacute phase (24–72 after the ischemic event) (Chen et al., 2019; Belov Kirdajova et al., 2020). Regarding the role of oligodendrocytes in stroke, available data indicate there is a substantial oligodendrocyte loss due to excitotoxicity and oxidative stress in the ischemic core; however, a significant increase in this cell population takes place within the penumbra (for a review see Jadhav et al., 2022). Finally, the chronic phase can extend for weeks after the initial damage, being probably caused by a delayed apoptotic neuronal death involving several factors like an uncontrolled inflammatory response, the persistent presence of neurotoxic/neuroinhibitory factors, and oxidative stress, among others (Allen and Bayraktutan, 2009; Zhang et al., 2012; Belov Kirdajova et al., 2020).

### Current Therapeutic Approaches to Ischemic Stroke

In recent years, several effective treatments have been developed for the treatment of ischemic stroke within the acute phase. Besides, the coordination between the emergency teams, neurologists, and hospital stroke units implementing what is known as “stroke code,” allows a faster intervention, which facilitates the patient’s admission to the stroke unit and administration of treatments that help reduce mortality and sequelae (Indredavik et al., 1991). Currently, the reperfusion therapies available for the treatment of ischemic stroke in the acute phase are intravenous (i.v.) thrombolysis and mechanical thrombectomy (Kuriakose and Xiao, 2020; Feske, 2021); however, due to the narrow therapeutic time window for effective intervention, less than 5% of patients can benefit from these treatments (Fernández-Ruiz et al., 2015; Choi et al., 2019). There are two types of thrombolytic treatments: i.v. injection of recombinant tissue plasminogen activator and tenecteplase, which are administered within the first 4.5 h after stroke onset. These treatments improve the patient’s clinical and functional outcome, evaluated within 3 months (Alonso de Leciñana et al., 2014). Another therapeutic option used when thrombolytic treatment cannot be administered or has not been effective is mechanical thrombectomy. There are several randomized studies that demonstrate the efficacy of this procedure when applied within 6 h of symptom onset (Powers et al., 2018).

With the aim of reducing sequelae and improving the functional evolution of patients, neuroprotective treatments are continuously under investigation (Miller et al., 2011; Kolb et al., 2019). Still, despite having promising results in animal models using neuroprotective drugs, these treatments have failed to improve neurological outcomes after ischemic stroke in clinical trials, probably because neuronal survival is not enough to promote brain recovery (Gleichman and Carmichael, 2014; Choi et al., 2019). For that reason, the study of glial cells as novel therapeutic targets in stroke has gained attention recently, not only because these cells have been demonstrated to be essential for proper brain functioning but also for their neuroprotective potential in different neurological pathologies, including stroke (Gleichman and Carmichael, 2014; Hersh and Yang, 2018; Jha et al., 2019).

### The Endocannabinoid System

The endocannabinoid system (ECS) constitutes an intercellular communication system that plays a fundamental role in the regulation of multiple physiological processes such as synaptic transmission, memory processes, nociception, inflammation, appetite, or thermoregulation, among others (Fernández-Ruiz et al., 2015; Cristino, Bisogno and Di Marzo, 2020; Estrada and Contreras, 2020). Consequently, the ECS and the elements that constitute it (receptors, endogenous ligands, and synthesis and breakdown enzymes) play a key role in neurotransmission, in the endocrine and the immune system.

Cannabinoids (CBs) exert their effects mainly via cannabinoid receptor 1 (CB_1_R) and cannabinoid receptor 2 (CB_2_R) (Matsuda et al., 1990; Munro et al., 1993). CB_1_R is widely expressed in the central nervous system (CNS), mostly in neurons but also in glial cells, while CB_2_R is characteristic of the immune system, being expressed as well by CNS cells like microglia, astrocytes and oligodendrocytes (Munro et al., 1993; Howlett, 2002; Molina-Holgado et al., 2002; Gulyas et al., 2004; Núñez et al., 2004; Benito et al., 2007; Navarrete and Araque, 2008; Turcotte et al., 2016; Fernández-Trapero et al., 2017). CBs also activate other receptors such as orphan G protein-coupled receptors (GPCRs), peroxisome proliferator-activated receptors (PPARs), or the adenosine A_2A_ receptor (A_2A_R) (Morales and Reggio, 2017; Franco et al., 2019; Iannotti and Vitale, 2021).

The main endogenous ligands or endocannabinoids (eCBs) are 2-arachidonoylglycerol (2-AG) (Mechoulam et al., 1995; Sugiura et al., 1995) and N-arachidonoylethanolamine or anandamide (AEA) (Devane et al., 1992). Both eCBs are synthesized “on demand” from membrane lipid precursors. 2-AG is the most abundant eCB and a full CB_1_R/CB_2_R agonist (Sugiura et al., 1995; Stella et al., 1997). It is synthesized by the enzyme diacylglycerol-lipase (DAGL) (Tanimura et al., 2010; Shonesy et al., 2015) and metabolized to arachidonic acid and glycerol by monoacylglycerol lipase (MAGL) (Dinh et al., 2002). By contrast, AEA is a partial CB_1_R agonist and it does not bind to CB_2_R (Silva et al., 2013; Zou and Kumar, 2018). AEA has a complex synthesis mechanism involving the action of the enzyme N-arachidonoyl phosphatidylethanolamine-phospholipase D (NAPE-PLD) (Di Marzo et al., 1994; Blankman and Cravatt, 2013), meanwhile, its degradation is carried out by the enzyme fatty acid amide hydrolase (FAAH), which metabolizes AEA to arachidonic acid and ethanolamide (Zou and Kumar, 2018).

### Role of the ECS in Ischemic Stroke

Similar to other neuropathologies, several studies have proved that the ECS is altered in ischemic stroke, as it has been reviewed elsewhere (Hillard, 2008; Fernández-Ruiz et al., 2015; Kolb et al., 2019; Cristino et al., 2020). However, contradictory and conflicting results have been found and to date, the role of the ECS in stroke has not been elucidated.

Endocannabinoid tone alterations have been reported in clinical studies and plasma levels of AEA were significantly elevated in samples from acute stroke patients (Schäbitz et al., 2002; Naccarato et al., 2010). Moreover, higher levels of 2-AG and other ECS-lipid mediators, such as palmitoylethanolamide (PEA), are positively correlated with neurological impairment (Naccarato et al., 2010). Very recently, an increased expression of CB_2_R and the microRNA miR-665, a potential CB_2_R regulator, were found in circulating monocytes of patients with acute ischemic stroke (Greco et al., 2021). These observations at the peripheral level could be reflecting disturbances at the central level, in line with those observed in postmortem tissues (Caruso et al., 2016), or suggesting its involvement in the modulation of the peripheral immune response in stroke patients (Greco et al., 2021).

The involvement of the ECS in the pathophysiology of stroke is even more evident in animal models (Schäbitz et al., 2002; Muthian et al., 2004; Zarruk et al., 2012; Sun, and Fang, 2013). In the transient middle cerebral artery occlusion (tMCAO) model using CB_1_R^−/−^ mice, a greater lesion volume was observed than in wild-type animals due to a decrease in CBF after reperfusion, probably due to a direct effect of CB_1_R activation on cerebrovascular smooth muscle cells (Hillard, 2000; Parmentier-Batteur et al., 2002). However, the administration of pharmacological treatments aimed at modulating CB_1_R function show controversial results. On the one hand, several works have shown that CB_1_R antagonism has neuroprotective effects in animal models of stroke (Muthian et al., 2004; Zhang et al., 2008; Schmidt et al., 2012; Knowles et al., 2016; Reichenbach et al., 2016). For example, the treatment with CB_1_R antagonists such as SR141716 (5 mg/kg) increases CBF in the affected brain area, decreases the lesion volume in both the tMCAO and the photothrombotic permanent MCAO (pMCAO) models, and improves the neurological function after stroke (Zhang et al., 2008; Reichenbach et al., 2016). In a rat model of global brain ischemia, the treatment with the CB_1_R antagonist AM251 (2 mg/kg) also shows neuroprotective effects on areas of the reward system, reducing neuronal death and improving behavioral test performance (Knowles et al., 2016). On the other hand, CB_1_R activation with the selective CB_1_R agonist ACEA, both after intracerebral and intraperitoneal (i.p.) administration (10 μM and 1 mg/kg, respectively), has also shown neuroprotective effects in the endothelin-induced MCAO (eMCAO) and pMCAO models, reducing neuronal death and brain injury volume (Schmidt et al., 2012; Caltana et al., 2015). Regarding the role of CB_2_R in ischemic stroke, there seems to be a greater consensus, since the majority of studies report a neuroprotective effect i.e., a reduction of infarct volume when CB_2_R agonists are administered in different animal models (Schomacher et al., 2008; Zhang et al., 2008; Reichenbach et al., 2016; Choi et al., 2018).

Although the role of the ECS in stroke may appear more complex than in other neurological pathologies, CB-based therapies begin to acquire special relevance for patients suffering from ischemic stroke (Kolb et al., 2019; Cristino et al., 2020). One of the main features of CB-based therapies is that they are multi-target molecules able to regulate the three main pathological mechanisms involved in neurodegenerative diseases, especially in ischemic stroke: inflammation, excitotoxicity, and oxidative stress. These effects could be mediated not only by CB_1_R and CB_2_R but other ECS-related receptors may also be involved, such as PPARγ or G-protein receptor 55 (GPR55) (Fernández-Ruiz et al., 2015; Aymerich et al., 2018). Although considerable progress has been made in the study of the ECS in neurons, there is also extensive evidence supporting the important modulatory role of the glial ECS for the proper function of these cells and their interactions with other cell types. However, the precise role of glial ECS remains a field barely explored but with great potential in stroke and other neurological pathologies (Egaña-Huguet et al., 2021; Jadhav et al., 2022).

## Microglia

Microglia, discovered in 1919 by the Spanish physician and histologist Pio del Rio-Hortega, constitute the first line of defense of the CNS (Mecha et al., 2016; Prinz, Jung and Priller, 2019). Microglia are highly dynamic cells included in the phagocytic-mononuclear cell lineage along with peripheral and CNS-associated macrophages (CAMS), monocytes, and dendritic cells (Ginhoux et al., 2010; Goldmann et al., 2016; Mecha et al., 2016; Prinz et al., 2019). However, there is currently an intense debate about its origin, since recent publications suggest that microglial cells originate in the yolk sac from myeloid progenitors during embryonic development whereas peripheral macrophages develop from hematopoietic stem cells (Prinz, Jung and Priller, 2019). After embryogenesis, microglia maintain a population of 5–20% of total glial cells in the mouse brain and around 0.5%–16.6% in the human brain by a process of self-renewal (Mittelbronn et al., 2001; Ginhoux et al., 2010; Askew et al., 2017). Long considered the macrophages of the brain, microglia have multiple functions in physiological and pathophysiological conditions. Very recently, it has been shown that mouse and human microglia also exhibit regional phenotypic heterogeneity (Böttcher et al., 2019), however, whether this heterogeneity correlates with a regional-specific function or if this is relevant for different pathologies, remains to be investigated.

**FIGURE 1:**
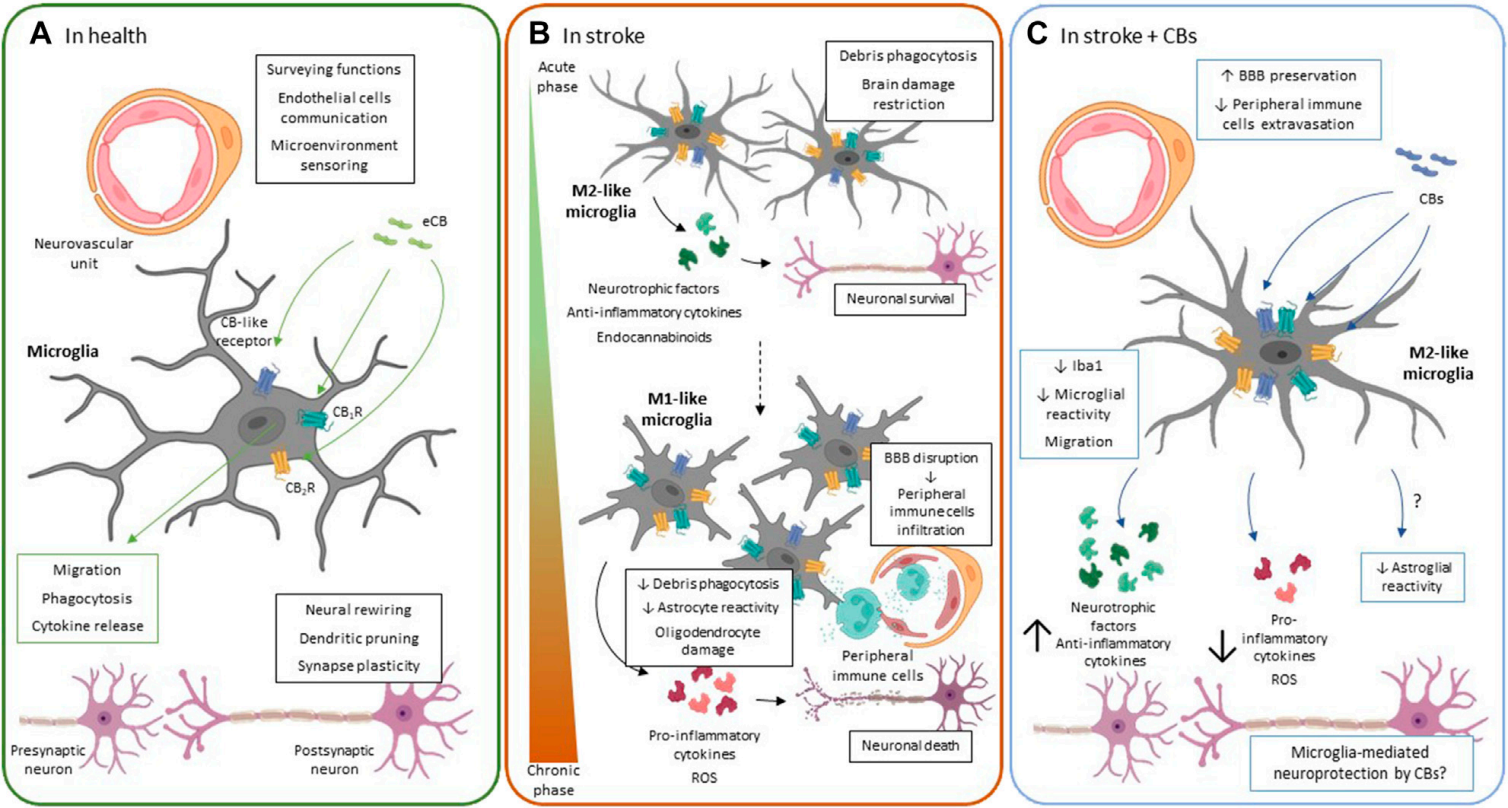
Microglial ECS function. **(A).** In healthy conditions, microglia participate in pivotal functions for proper neuronal functioning such as dendritic pruning, neural rewiring, secretion of trophic factors and synaptic plasticity. The main function of resting microglia is to monitor the brain parenchyma through their widely branched morphology and to quickly detect any type of cellular alteration or damage. Cannabinoid receptor activation regulates the phenotypic polarization of microglia, migration, cytokine production and phagocytic capacity of these cells. **(B).** Microglial response to ischemic stroke follows a spatio-temporal pattern. Initially, in the acute phase, there is a significant increase in the number of M2-like microglial cells in the ischemic area. The M2-like phenotype is considered protective since it acquires phagocytic capacity that allows it to eliminate the dead cells debris. In addition, they release neurotrophic factors and anti-inflammatory cytokines in an attempt to limit neuronal damage. However, during the chronic phase of stroke M1-like cells proliferate and are recruited to the injury area. M1 microglia release pro-inflammatory cytokines and ROS that contribute to exacerbate neuronal death, oligodendrocyte damage and astrocyte activation. The inflammatory response also contributes to BBB rupture and the release of chemoattractant factors from peripheral immune cells. The M1 phenotype is characterized by the acquisition of an amoeboid morphology, without branching and losing its phagocytic capacity thus preventing tissue repair. The expression of CB_1_R and CB_2_R as well as other ECS-related receptors has been upregulated in different *in vitro* and *in vivo* stroke models. However, its specific role in microglia is still unknown. **(C).** The protective effects observed after treatment with CBs in different stroke models include modulation of microgliosis. Reduction in the number of microglia cells was not only observed, but also induced polarization towards the M2-like phenotype. The M2-like microglia contributes to tissue repair, as it has phagocytic capacity and releases trophic factors. In addition, it releases anti-inflammatory cytokines limiting brain damage and preserving the BBB, thus decreasing peripheral immune cell extravasation. Green arrows and boxes: eCB-mediated effects; blue arrows and boxes: CB-mediated effects. BBB: brain-blood barrier; CB_1_R: cannabinoid receptor 1: CB_2_R: cannabinoid receptor 2; CBs: cannabinoids; eCBs: endocannabinoids; ROS: reactive oxygen species.

Under physiological conditions, microglial cells participate in important functions like dendritic pruning, neural rewiring, oligodendrocyte precursor cells (OPCs) differentiation, and synaptic plasticity, among other cellular processes (Figure 1A) (Nakajima et al., 2001; Wake et al., 2009; Matcovitch-Natan et al., 2016; Filipello et al., 2018). *In vivo* imaging studies have demonstrated that resting microglia show a small soma with highly dynamic branching morphology, acting as sensors that detect changes in the brain parenchyma. Following an acute injury, microglia are activated, changing their morphology to an amoeboid shape and modifying their branching pattern that rapidly are directed towards the lesion site (Davalos et al., 2005; Nimmerjahn et al., 2005). Changes also occur at the molecular level, including epigenetic, transcriptomic, and proteomic modifications (Mecha et al., 2015; Prinz et al., 2019). Moreover, once activated after a harmful event in the CNS, microglia undergo the process of phenotypic polarization, shifting toward one of two main opposite phenotypes: the classically activated pro-inflammatory state (M1-like) or the alternative anti-inflammatory protective state (M2-like). In addition, similarly to the phenotypic classification of macrophages, within the M2-like phenotype, different microglial subtypes (M2a, M2b, and M2c) have been associated with repairing, immunoregulatory, or deactivating phenotype functions, respectively (Zawadzka et al., 2012; Mecha et al., 2016; Kanazawa et al., 2017). However, because most studies have been performed in cell culture, further *in vivo* studies are needed to establish not only the function of the different subtypes of the microglial M2-like phenotype, but even their existence in the pathophysiological context, as recently reviewed by Tanaka et al. (2020).

The phenotypic polarization of microglial cells to M1 upon stimulation with bacterial-derivative molecules such as lipopolysaccharide (LPS) or even interferon gamma (IFN-γ) has been characterized *in vitro*. Under these conditions, M1 cells release a wide variety of pro-inflammatory cytokines and chemokines like TNF-α, IL-1α, IL-1β, IL-6, or IL-12 (Michelucci et al., 2009; Zawadzka et al., 2012; Malek et al., 2015; Mecha et al., 2015). Polarization to M1 also induces the expression of genes such as iNOS, ROS production, and the activation of the inflammasome complex (Shi et al., 2012; Gong et al., 2018). Stimulation with anti-inflammatory cytokines, i.e. IL-4 or IL-10 promotes a polarization toward the M2-like phenotype (Michelucci et al., 2009; Lively and Schlichter, 2013). Other cytokines and certain chemokines, including IL-3, IL-21, CCL2, and CXCL4, also induce M2 polarization. M2 cells in turn release anti-inflammatory cytokines such as IL-4, IL-10, IL-13, or TFG-β (Michelucci et al., 2009; Mecha et al., 2015).

The role of the different microglial phenotypes has been the subject of intense study in recent years and is becoming increasingly relevant given the dual functions of these cells in pathophysiological processes associated with acute and chronic diseases, including ischemic stroke (Kanazawa et al., 2017; Qin et al., 2019).

### Microglial Function in Ischemic Stroke

The contribution of microglia to the neuroinflammatory context of ischemic stroke is controversial, as microglial cells could exert both detrimental and beneficial effects (for review consult Qin et al., 2019). On the one hand, post-ischemic inflammation has been considered a negative factor that worsens patient outcome, since activated microglia carry out striping processes that disrupt synaptic connections resulting in the functional impairment of neuronal circuits after ischemic damage (Wake et al., 2009). But on the other hand, it seems to be a necessary process for the clearance of cellular debris and dead cells through phagocytosis and to trigger repairing processes that promote functional brain recovery (Ma et al., 2017; Qin et al., 2019; Rajan et al., 2019).

Following an ischemic stroke, activated microglial cells change their morphology and rapidly migrate to the focus of injury as they are sensitive to fluctuations in blood flow and respond to BBB rupture and to cell death occurring in the acute phase of stroke (Nimmerjahn et al., 2005; Masuda et al., 2011; Ju et al., 2018). Besides, in response to damage, CNS resident microglia continuously proliferate, contributing with new cells to the resident microglial pool (Li et al., 2013). The extravasation and migration of peripheral immune cells (Iadecola et al., 2020), as well as the mobilization of pericytes close to the injury, also increase the microglial pool site (Özen et al., 2014; Roth et al., 2019). Despite monocyte extravasation in stroke has recently gained a considerable amount of interest, the specific contribution of individual cell types to the progression or repair of ischemic damage is still under intense study (Urra et al., 2009; Rajan et al., 2019). Overall, the microglial response to ischemic stroke is very complex and follows a spatio-temporal pattern. First, studies in animal models of both permanent and transient ischemia, have demonstrated a dramatic increase in microglial cells between 24 h and 7 days post-ischemia (Michalski et al., 2012; Li et al., 2013; Cotrina et al., 2017; Rajan et al., 2019). This peak in cell number in the ischemic core appears later in models of photothrombotic ischemia, being a slightly more moderate response (Li et al., 2013; Cotrina et al., 2017). Furthermore, in a tMCAO model, M2-like microglia is greatly increased in the ischemic zone, probably as an immediate response to neuronal damage that tries to eliminate cellular debris and limiting the extent of tissue damage (Figure 1B) (Hu et al., 2012). Soon after microglial activation in the pMCAO model, phagocytic microglial cells enclosing MAP2-positive neurons are observed (Cotrina et al., 2017). However, this context changes during the chronic phase of stroke in which M1 microglial cells proliferate and are recruited. M1 microglia release pro-inflammatory cytokines and ROS that contribute to exacerbating neuronal death, BBB breakdown, and also have a reduced phagocytic capacity that prevents tissue repair (Figure 1B) (Hu et al., 2012; Chen et al., 2019). Phenotypic changes of microglia are also region-specific, with amoeboid-shaped cells being located in the core and penumbra of the lesion and less branched cells in the peri-infarct zone (Li et al., 2013; Cotrina et al., 2017; Rajan et al., 2019).

Activated microglia orchestrate the response to ischemic damage by communicating not only with neurons but also with non-neuronal cells and BBB structural components (Mecha et al., 2016; Huang et al., 2020). In fact, the interaction between activated microglia and astrocytes plays a crucial role in the process of neuroinflammation. The release of cytokines and trophic factors by microglia promotes phenotypic change in astrocytes, thus, M1 microglia releases, among other factors TNF-α, IL-1β or C1q, favoring the neurotoxic reactivity state of astrocytes (Liddelow et al., 2017). This communication is bidirectional, such that astrocytes also influence microglial phenotypic changes in a neuroinflammatory context by secreting a wide range of chemokines (for review, Jha et al., 2019; Liu et al., 2020). Besides, activated microglia also interact with oligodendrocytes, with a vast amount of data suggesting a deleterious effect of M1 microglia and the pro-inflammatory cytokines they release, on oligodendrocyte survival (Moore et al., 2015; Fan et al., 2019). On the other hand, in multiple sclerosis models, an increased differentiation of OPCs and, therefore, activation of remyelination processes favored by M2-like microglia has been described (Miron et al., 2013). This oligodendrocyte-protective effect has also been observed in an animal model of bilateral common carotid artery stenosis, where the treatment with the immunomodulatory drug Fingolimod promotes the polarization of microglia towards the M2-like phenotype leading to increased survival of OPCs and favoring myelin repair processes (Qin et al., 2017). In light of the complex microglial response in stroke and the dual effect of the phenotypes described, the search for new therapeutic options with a modulating effect on cell polarization in stroke has intensified in recent years (Qin et al., 2017; Liu et al., 2019; Lu et al., 2021).

### Microglia and the ECS

Under physiological conditions, both in animals and humans, microglia hardly express CB_1_R and CB_2_R (Benito et al., 2007; López et al., 2018; Egaña-Huguet et al., 2021). However, numerous studies show that the expression pattern of both receptors are altered in microglial cells in neuropathological conditions, e.g., Alzheimer’s disease (Benito et al., 2003; López et al., 2018), multiple sclerosis (Benito et al., 2007), Down’s syndrome (Núñez et al., 2008), spinocerebellar ataxia (Rodríguez-Cueto et al., 2014), immunodeficiency virus infection (Benito, 2005) or Huntington’s disease (Palazuelos et al., 2009).

In general, in an *in vivo* neuroinflammatory context, an increase in CB_2_R levels is associated mostly with the presence of microglia around neuropathological hallmarks, e.g., protein aggregates (Benito et al., 2007; Núñez et al., 2008; López et al., 2018). However, *in vitro* studies show the complexity of the microglial response since microglial activation and polarization seem to vary depending on the stimulus used, the manipulation of the cell culture, or even the intrinsic heterogeneity of these cells (Pietr et al., 2009; Mecha et al., 2016; Gosselin et al., 2017). A few years ago, Mecha and others demonstrated changes in the different constituents of the ECS when microglia polarization proceeds *in vitro*. The classical activation of rodent microglia with LPS induces a downregulation not only of CB_1_R and CB_2_R but also of the eCB synthesis and degradation enzymes (Malek et al., 2015; Mecha et al., 2015). By contrast, alternative activation, which polarizes microglia towards the M2-like phenotype, upregulates the expression of CB_2_R and the eCB synthesis enzymes. Consequently, M2 microglia are able to produce and release 2-AG and AEA in greater quantities than in the resting state (Walter et al., 2003; Mecha et al., 2015). However, the use of other stimuli, such as IFNɣ, does not seem to influence the expression of CB_2_R (Carlisle et al., 2002; Maresz et al., 2005). Finally, activation of CB_2_R regulates pivotal functions of microglia, such as their migration capacity (Walter et al., 2003; Guida et al., 2017), phagocytosis (Tolón et al., 2009), and cytokine release (Malek et al., 2015) (Figure 1A).

Regarding CB_1_R, it has been recently demonstrated that the human microglial cell line N9 expresses this receptor in the resting state. Although no changes in CB_1_R expression levels are detected after stimulation with LPS and IFNɣ, proximity ligation assays show that CB_1_R-CB_2_R heterodimers are formed following the inflammatory stimulus (Navarro and Borroto-Escuela, 2018). Another study has shown that despite low CB_1_R expression, the treatment with the selective CB_1_R antagonist SR141716A (1 μM) induces the polarization of BV-2 microglia towards the M1 phenotype. Moreover, the use of this antagonist prevents the anti-inflammatory effects of the non-selective cannabinoid agonist, WIN55,212-2 (1 μM) (Lou et al., 2018). All these data could suggest the involvement of CB_1_R in microglial polarization and function, raising the possibility of its pharmacological manipulation to modulate the inflammatory response in neurological diseases.

Microglia also express other non-CB receptors through which certain CBs can exert their effects. This is the case of PPARs, which seem to play a relevant role in microglial polarization (Ji et al., 2018). In particular, PPARα can be activated *in vitro* by the eCB-like compound PEA, leading to an increase in CB_2_R expression and 2-AG production (Guida et al., 2017), and the migration capacity of these cells (Franklin et al., 2003; Guida et al., 2017). The orphan receptor GPR55 is also expressed in microglia and is attracting special attention in different neuroinflammatory pathologies (Marichal-Cancino, 2017; Saliba et al., 2018; Burgaz et al., 2021). This receptor appears to follow a similar expression pattern to CB_2_R when microglia are stimulated with LPS. However, differences have been observed between the use of cell lines and primary microglial cultures. These differences are probably due to an intrinsic heterogeneity in the microglia cell lines used, or even to the possibility that in primary cultures, microglia are in a primed state due to the handling necessary for the development of *in vitro* assays (Pietr et al., 2009; Saliba et al., 2018). GPR55 blockage has anti-inflammatory effects by reducing prostaglandin production by microglia following LPS stimulation (Saliba et al., 2018). Finally, GPR55 activation by 2-AG or the synthetic cannabinoid abnormal-cannabidiol (abn-CBD), promotes BV-2 cell activation and migration (Franklin and Stella, 2003; Walter et al., 2003; Ryberg et al., 2007).

### Microglial ECS Pharmacological Modulation in Stroke

Although the molecular mechanisms involved in CB-based neuroprotection are still not known in detail, we do know that microglia and microglial ECS play a relevant role in stroke. Due to the role of CB_2_R on microglial migration and polarization and also on extravasation of peripheral immune cells to the CNS, the study of this receptor has received a great interest in stroke (Zhang et al., 2008; Hosoya et al., 2017; Greco et al., 2021). Several reports have shown an increased expression of CB_2_R in the ischemic penumbra in tMCAO, eMCAO, and also in both adult and neonatal hypoxia-ischemia (HI) murine models (Ashton et al., 2007; Zhang et al., 2008; Fernández-López et al., 2012; Schmidt et al., 2012). This upregulation occurred in macrophage-like cells that could be resident microglia or infiltrated peripheral monocytes (Ashton et al., 2007; Schmidt et al., 2012). A more recent study, using a model of photothrombotic ischemia combined with positron emission tomography and histological techniques, describes an early increase in CB_2_R expression in the peri-infarct area that colocalizes with some microglial cells showing amoeboid morphology. They even detect CB_2_R in branched microglia of the contralateral hemisphere that could be in a primed state, highlighting the role of this receptor in the different states of microglial activation (Hosoya et al., 2017). Pharmacological CB_2_R activation using selective agonists leads to a reduction in lesion volume and cognitive improvement in different animal models of stroke (Ashton et al., 2007; Zhang et al., 2008; Schmidt et al., 2012; Ronca et al., 2015). In addition, improved regional microcirculation in the affected area, decreased leukocyte rolling and extravasation, and preserved BBB integrity (Zhang et al., 2007, 2008). The involvement of microglial CB_2_R in BBB preservation has also been studied in animal models of intracerebral hemorrhage and traumatic brain injury. In these studies, selective CB_2_R activation reduces the release of pro-inflammatory cytokines by microglia and upregulates the expression of molecules necessary for the maintenance of tight junctions such as zo-1 or claudin-5, which are essential for BBB integrity (Figure 1C) (Amenta et al., 2012; Li et al., 2018). Recently, the protective and neuroinflammatory-modulating potential of β-caryophyllene (BCP), a terpene derived from *Cannabis sativa*, which acts as a CB_2_R agonist, has been demonstrated. In a model of photothrombotic ischemia, the treatment with BCP alone or in combination with cannabidiol (CBD), the main non-psychoactive constituent of *Cannabis sativa*, reduced the infarct area in a dose-dependent manner, and modulated both the number and morphology of microglial cells (Yokubaitis et al., 2021).

CB_1_R expression is also altered in stroke patients and in animal models (Zhang et al., 2008; Schmidt et al., 2012; Caltana et al., 2015; Caruso et al., 2016). A study performed in postmortem samples from patients revealed increased CB_1_R immunohistochemical labeling in the ischemic area. This pattern was associated both with neuronal and non-neuronal cells suggesting a role of CB_1_R in the glial neuroinflammatory response following acute ischemic damage (Caruso et al., 2016). However, the use of CB_1_R agonists and antagonists in different animal models of stroke have shown controversial results as previously explained. These results could be explained by the diversity of animal models used that may affect differently the receptor’s abundance after ischemic injury, to CB_1_R desensitization effects depending on the ligand used and the dose, or even by the different roles played by this receptor depending on the cell type where it is expressed. To date, little is known about the specific role of CB_1_R in microglia in the context of ischemic stroke. In the tMCAO model, an early and modest increase in CB_1_R expression has been described in microglia after ischemic damage in the ipsilateral hemisphere (Schmidt et al., 2012). Moreover, CB_1_R activation by 1 mg/kg i.p. administration of ACEA in a pMCAO model, not only reduces lesion volume, but also reduces glial reactivity, by decreasing the number of lectin-positive cells. Notably, it also reduced the number of microglial cells with amoeboid morphology in favor of cells with a more branched morphology, both in the short and long term (Caltana et al., 2015). These data, together with those previously mentioned (Lou et al., 2018; Navarro and Borroto-Escuela, 2018), indicate that the study of this receptor and its function in relation to the phenotypic polarization of microglia should be further explored.

In recent years, CBD has gained special importance in the context of ischemic brain injury (Hayakawa et al., 2010; Fernández-Ruiz et al., 2015; Mori et al., 2017, 2021; Martínez-Orgado et al., 2021; Yokubaitis et al., 2021; Khaksar et al., 2022). CBD is a multitarget molecule with a complex pharmacology. Although it initially showed a low affinity for CB receptors, it has subsequently been shown that it can act as an antagonist of CB_1_R and CB_2_R at low concentrations (Pertwee, 2008; Navarro and Reyes-Resina, 2018). Noteworthy, CBD also has an affinity for other ECS-related receptors such as GPR55, 5-HT_1A_, TRPA1, TRPV1-4 or PPARɣ (Pertwee, 2008; Britch et al., 2021). In the different animal models of stroke used, it has been shown that CBD treatment improves the motor deficits observed after ischemic damage and reduces the area of injury (Hayakawa et al., 2004; Schiavon et al., 2014; Ceprián et al., 2017; Mori et al., 2017). In adult animals, CBD facilitates neuroplasticity after tMCAO by decreasing glial reactivity, reducing both the number of reactive microglia and astrocytes in the hippocampus, and favoring the release of neurotrophic factors, such as brain-derived neurotrophic factor (Schiavon et al., 2014; Mori et al., 2017). Interestingly, neuroprotective effects of CBD have also been observed in a neonatal HI stroke model, both in the short and long term. Besides, there is an improvement in the performance of motor tests despite the fact that no decrease in lesion volume is observed. In the same study, administration of 5 mg/kg of CBD, also decreased glial reactivity, decreasing the number of microglial cells in the ipsilateral hemisphere (Ceprián et al., 2017). These results are similar to those obtained in neonatal models of HI in piglets and in rodents. In those studies, 1 mg/kg of CBD, shows neuroprotective effects by decreasing neuronal death and anti-inflammatory effects by modulating cytokine release and decreasing the number of reactive astrocytes and microglia after HI injury (Figure 1C) (Lafuente et al., 2011, 2016; Pazos et al., 2012, 2013; Mohammed et al., 2017; Barata et al., 2019). CBD appears to modulate microglial polarization by promoting a less amoeboid and more branching phenotype (Mohammed et al., 2017; Barata et al., 2019). Several pieces of evidence demonstrate the multitarget effect of CBD in stroke and/or HI models involving the CB_1_R, CB_2_R, GPR55, 5-HT_1A_, and PPARɣ receptors (Mishima et al., 2005; Castillo et al., 2010; Pazos et al., 2013; Mori et al., 2021). Since microglia express most of these receptors, this strengthens the idea of the important role played by the ECS in the polarization, cell renewal and migration of microglia particularly in the context of stroke. However, there are still many unknowns about the precise role of the ECS in microglial polarization and function and the molecular mechanisms involved in those processes, which must be addressed to find new CB-based therapies for stroke treatment.

## Astrocytes

**FIGURE 2:**
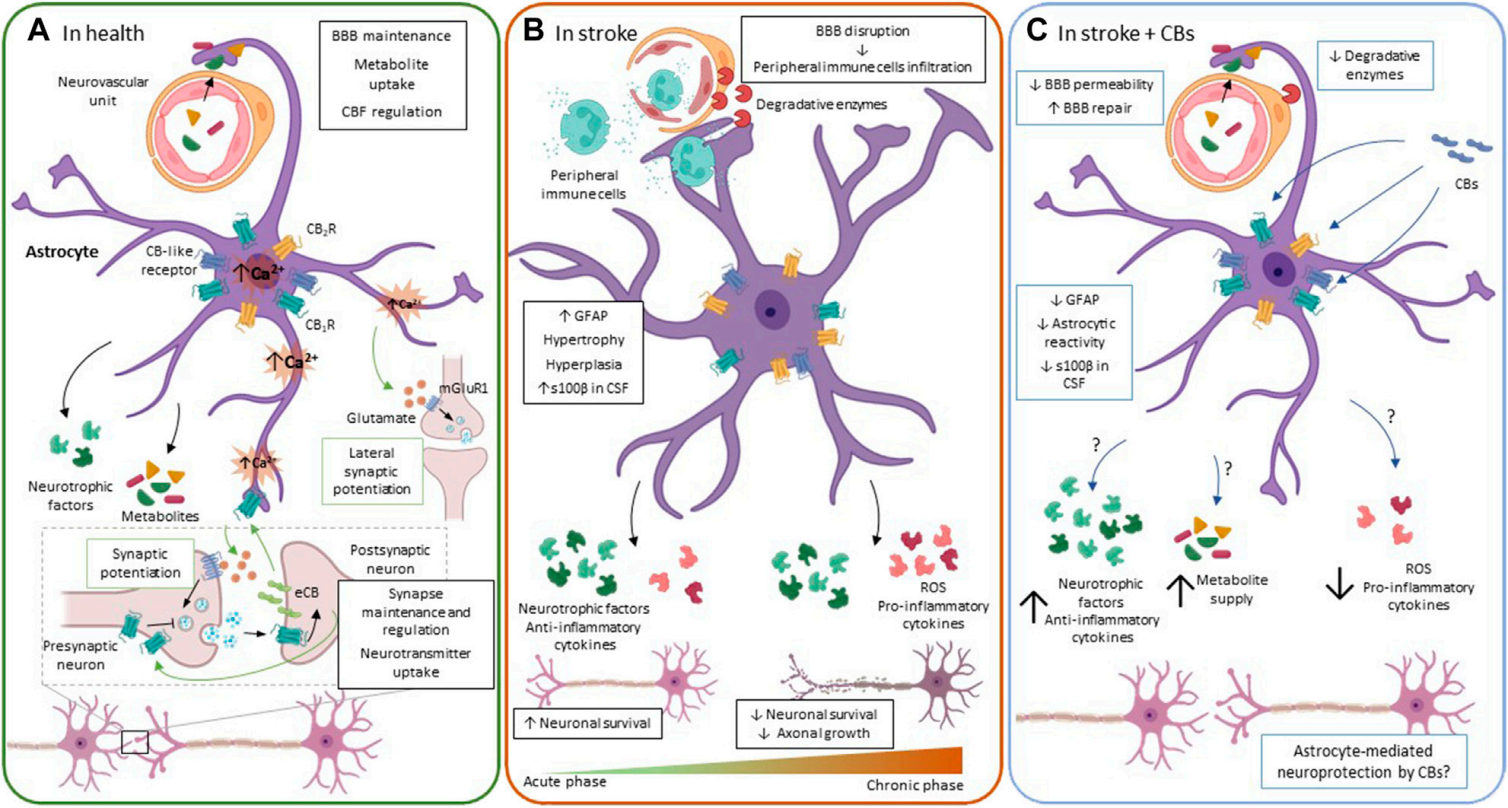
The ECS functioning in astrocytes. **(A).** In physiological conditions, astrocytes regulate processes that are crucial for neuronal functioning, e.g., providing metabolic and trophic support for neurons, controlling CBF, BBB permeability, and regulating synapse maintenance and plasticity, among others. Astrocytes participate in synapse plasticity through the tripartite synapse. When neurotransmitter is released by presynaptic neurons, it increases intracellular Ca^2+^ both in post-synaptic terminals and in astrocytes. In post-synaptic neurons, Ca^2+^ elevation induces eCB release to the extracellular space, inhibiting neurotransmitter release from the presynaptic neuron. In astrocytes, eCB binding to their receptors mobilize Ca^2+^ from internal stores, triggering gliotransmitter release, i.e., glutamate, which in turn promotes mGluR1-mediated neurotransmitter release from the presynaptic neuron, a phenomenon called synaptic potentiation. Besides, as Ca^2+^ spreads intracellularly in the astrocyte, it is able to release glutamate at distal points, stimulating synapsis that are at a certain distance from the synapse that was initially stimulated. This results in the so-called lateral synaptic potentiation. **(B).** During the acute/subacute phase after an ischemic event, astrocytes undergo significant morphological and functional changes like hypertrophy, hyperplasia and increased GFAP levels. To increase neuronal survival, astrocytes release neurotrophic factors and anti-inflammatory cytokines. Nevertheless, they also release pro-inflammatory cytokines that negatively affect neuronal survival. Moreover, at the neurovascular unit, astrocytes upregulate the expression of surface receptors and enzymes that are strongly associated with inflammatory responses that actively contribute to BBB disruption and leukocyte infiltration into the CNS, becoming a source of inflammation if this process becomes chronic. Over time, reactive glial cells rearrange, creating a barrier composed of densely packed cells that separate the ischemic core from the penumbra. **(C)**. Main molecular changes induced in astrocytes by CBs after stroke. CB modulation of astrocyte reactivity includes reduced GFAP immunoreactivity and release of catalytic enzymes, leading to attenuation of BBB disruption. These molecules also increase neuronal survival after ischemia; however, it remains unclear whether the neuroprotection exerted by CBs is mediated by astrocytes. Green arrows and boxes: eCB-mediated effects; blue arrows and boxes: CB-mediated effects. BBB: brain-blood barrier; CB_1_R: cannabinoid receptor 1: CB_2_R: cannabinoid receptor 2; CBs: cannabinoids; CSF: cerebrospinal fluid; eCB: endocannabinoids; ROS: reactive oxygen species.

Astrocytes are one of the most numerous cell populations in the CNS, where they exert many crucial homeostatic functions that allow the development and proper function of this system and the brain cells (Sofroniew and Vinters, 2010; Clarke and Barres, 2013; Verkhratsky and Nedergaard, 2018). These functions include the regulation of extracellular concentrations of ions and neurotransmitters, the formation and elimination of synapses, cytokine and neurotrophin secretion, CBF and metabolism regulation, among others (Figure 2A) (Sofroniew, 2020). Classically, astroglial cells have been classified in two different groups according to their location and morphology with protoplasmic astrocytes located mainly in gray matter, and fibrous astrocytes predominantly found in white matter (Miller and Raff, 1984). However, over the last years, increasing evidence has changed this conception and now it is acknowledged that astrocytes are highly heterogeneous, exhibiting important morphological and physiological differences among brain regions and significant differences in gene expression and protein content (Molofsky et al., 2012; Höft et al., 2014; John Lin et al., 2017; Miller, 2018). Thus, astrocyte functions vary depending on the neural populations they are associated with and/or the biological environment surrounding them. Moreover, astrocyte heterogeneity is species-dependent, with higher morphological and possibly functional complexity in the human brain compared to rodents (Kettenmann and Verkhratsky, 2008). In the healthy brain, astroglial cells provide structural support for neurons, actively participating in the regulation of neuronal growth and synapse formation, maturation, maintenance, and pruning (Sofroniew and Vinters, 2010; Clarke and Barres, 2013). They also play an active role in synaptic transmission, by being part of the tri-partite synapse, they support neuronal signaling, neurotransmitter uptake regulation, gliotransmitter and calcium release, modulating in this way synaptic plasticity and learning (Pereira and Furlan, 2010; Sofroniew and Vinters, 2010; Verkhratsky et al., 2012). One important function of astrocytes is their involvement in the maintenance and functionality of the BBB, particularly via astrocyte endfeet, together with endothelial cells and pericytes (Alvarez et al., 2013; Siqueira et al., 2018). Astrocytes are responsible for the selective diffusion of molecules through the BBB, allowing ion diffusion and regulating the entry of small molecules and water to the CNS. At the same time, these cells regulate the supply of oxygen and nutrients to neurons by taking up glucose, lactate, or ketone bodies from the bloodstream and transferring them to neurons as a source of energy, and/or by releasing trophic factors that are essential for neuronal survival (Rouach et al., 2008; Alvarez et al., 2013; Dezonne et al., 2013; Sotelo-Hitschfeld et al., 2015; Benarroch, 2016). In addition, these cells directly regulate BBB function by releasing molecules i.e. sonic hedgehog, nitric oxide, and vascular endothelial growth factor, which are involved in tight junction development, vasodilation, and angiogenesis (Nian et al., 2020)**.**

In homeostatic conditions, astrocytes are in a quiescent or resting state and become reactive in response to different stimuli or insults to the CNS like infections, trauma, neurodegenerative diseases, and stroke (Moulson et al., 2021). Astrocyte reactivity is in the first instance a physiological response that involves phenotypic and molecular changes aimed at restoring homeostasis and neurological function through diverse mechanisms (Sofroniew, 2020). However, in pathological conditions, these cells have biphasic functions, being beneficial or detrimental through cell-autonomous or non-cell-autonomous mechanisms, depending on the biological context. For example, if the initial insult is not resolved and becomes chronic, astrocytes can contribute to exacerbating the damage either by losing/gaining functions (Sofroniew, 2020). Recently, it was demonstrated the existence of at least two different types of reactive astrocytes (Zamanian et al., 2012; Liddelow et al., 2017). Under neuroinflammatory conditions, astrocytes polarize toward an A1-neurotoxic reactivity state, expressing different pro-inflammatory proteins and possibly other toxic molecules that induce synapse loss and neuronal death, whilst A2-neuroprotective reactive astrocytes are induced after an ischemic insult and promote neuronal survival (Liddelow et al., 2017; Guttenplan et al., 2021). Nevertheless, late discoveries on regional and local heterogeneity of astrocytes are shedding light on their complex developmental, morphological, molecular, physiological, and functional diverseness, modifying this dual classification of astrocytes (for a review on this topic see (Pestana et al., 2020). Newer hypotheses sustain the existence of mixed populations (subtypes) of astroglial cells that coexist in the resting state with a continuum in the intensity of reactivity states (Miller, 2018; Khakh and Deneen, 2019; Pestana et al., 2020; Sofroniew, 2020). Thus, the existence of different astrocyte subtypes in the resting state could explain different responses to the same insult, resulting in a variety of reactive states, an idea supported by data from various neurodegenerative disease models (Clarke et al., 2018; Yun et al., 2018; Smith et al., 2020). As the knowledge of astrocyte biology is constantly evolving due to the development and availability of new tools/experimental approaches, like single-cell transcriptomics, which provides valuable data on astrocyte heterogeneity and reactivity (Moulson et al., 2021), we could expect more refined and possibly unified concepts in the forthcoming years, as recently discussed by Escartin et al. (2021).

### Astrocyte Function in Ischemic Stroke

Similar to what occurs with other CNS cells, astrocytes undergo significant morphological, molecular, and functional modifications after an ischemic event (Figure 2B). These changes are very dynamic and rely not only on astrocytes but also on interactions and intercommunication with other CNS cells, notably neurons, microglia, and oligodendrocytes. In pathological circumstances like stroke, injured neurons and other cells communicate with astrocytes by releasing cytokines and other molecules, triggering astrocyte activation and causing profound changes in the synthesis and expression of other molecules (Sofroniew, 2009; Scarisbrick et al., 2012). After the stroke, there is a massive response of astrocytes, called reactive astrogliosis, but the timeline of astroglial activation is slower than in neurons or in microglia (Revuelta et al., 2019). Reactive astrogliosis is characterized by an increased expression of the glial fibrillary acidic protein (GFAP) and changes in cell morphology like hyperplasia and hypertrophy (Sofroniew, 2009; Scarisbrick et al., 2012; Kozela et al., 2017). Moreover, activated astrocytes release pro-inflammatory cytokines, like IL-1β, IL-6, and TNF-⍺, modulating the immune response and actively participating in the inflammatory process initiated after an ischemic event (Zamanian et al., 2012). Reactive astrocytes also synthesize and release some anti-inflammatory cytokines and neurotrophic factors that protect neurons, enhance neuronal synapses and plasticity, and improve functional outcomes after the stroke (Swanson et al., 2004; Li et al., 2008; Cekanaviciute et al., 2014). Noteworthy, astrocyte response after an ischemic event will significantly depend on the astrocyte subtype and possibly on the brain region affected. On the one hand, proliferative reactive astrocytes will increase their number and form limitant borders surrounding the infarcted area, constituting in conjunction with other cells a physical barrier around the necrotic tissue in the brain. These limitant borders allow setting boundaries to the damaged area, releasing molecules that promote neuronal growth and survival, and avoiding the spreading of neuroinflammation (Swanson, Ying and Kauppinen, 2004; Li et al., 2008; Huang et al., 2014; Sofroniew, 2020). However, these densely packed reactive astrocytes are also considered a source of pro-inflammatory molecules, ROS, and neurotoxicity that ultimately inhibit axonal regeneration (Gris et al., 2007). On the other hand, nonproliferative reactive astrocytes acquire diverse reactivity states. In contrast to microglial cells, which are very mobile, these astrocytes do not migrate from the penumbra to the ischemic core, instead, they polarize their processes to be able to exert their phagocytic abilities (Huang et al., 2014), having as well the ability to change their gene expression pattern and functions depending on their particular context. If the acute initial insult is not resolved and becomes chronic, nonproliferative reactive astrocytes can contribute to exacerbating the damage either by losing/gaining functions, as mentioned lines above (Sofroniew, 2020).

Another major event that occurs after stroke is BBB integrity disruption (Fernández-López et al., 2012; Arba et al., 2017), which favors ROS generation, the infiltration of inflammatory cells like leukocytes, and the production of proteolytic enzymes that ultimately exacerbate brain edema and neuroinflammation (Figure 2B) (Fernández-López et al., 2012; Arba et al., 2017). The BBB is constituted by endothelial cells, pericytes, and astrocytes. Some evidence indicates that changes in astrocytic proteins involved in BBB maintenance like metalloproteinase-2 and the toll-like receptor 4/metalloproteinase-9 (TLR4/MMP9) signaling pathway are upregulated after stroke, contributing to the disruption of astrocyte-endothelial junctions, consequently altering BBB permeability (Liu et al., 2017; Rosciszewski et al., 2018).

There are apparently contradictory roles of reactive astrocytes after stroke regarding the extent of their putative toxic or protective effects that could be difficult to measure, but several studies using GFAP^−/−^ mice in models of stroke and acute trauma are shedding some light on this matter (Nawashiro et al., 2000; Wilhelmsson et al., 2004; Li et al., 2008). For example, GFAP^−/−^ mice showed an impaired physiological response to ischemia in the pMCAO with transient carotid artery occlusion (CAO) model (Nawashiro et al., 2000), and GFAP^−/−^Vimentin^−/−^ mice had a higher infarct area and decreased glutamate transport by astrocytes than wild type mice after MCA transection (Li et al., 2008). Besides, GFAP^−/−^Vimentin^−/−^ mice subjected to an acute entorhinal cortex lesion model showed an attenuated astrocyte reactivity response, evidenced by fewer processes and dysregulation of endothelin B receptors, which allowed synaptic recovery in the hippocampus, changes that were associated with improved post-traumatic regeneration (Wilhelmsson et al., 2004).

Recently, Rakers and co-authors explored in more detail astrocyte reactivity in mice subjected to the tMCAO stroke model and found an upregulation of the canonical markers of reactive astrogliosis, the so-called pan-reactive transcripts, and a prominent increase of A2-reactivity specific transcripts (Rakers et al., 2019). Their observations suggest that these A2-like reactive astrocytes protect neurons and promote neuroregeneration after stroke. Moreover, they also observed significant changes in the expression of genes related to extracellular matrix composition, cell migration, cell-cell adhesion, and glial scar formation, further indicating that A2-astrocytes may help contain and restrict neuroinflammation and support neuronal survival (Rakers et al., 2019). Nevertheless, among the upregulated genes they found were several genes related to neuroinflammation, the complement cascade, apoptosis, and leukocyte transendothelial migration. Thus, they observed the coexistence of genes with potentially neurotoxic and neuroprotective functions in astrocytes from brain homogenates of mice subjected to tMCAO. Whether this phenomenon is due to the presence of astrocytes with a spectrum of different phenotypes that vary from neuroprotective to neurotoxic, or due to the activation of neuroprotective and neurotoxic signaling pathways within individual astrocytes remains to be determined.

### Astrocytes and the ECS

The presence of CB_1_R, CB_2_R, and other CB-like receptors has been demonstrated in astrocytes (Pazos et al., 2005; Sheng et al., 2005; Navarrete and Araque, 2008; Stella, 2010; Yang et al., 2019; Cristino et al., 2020). Besides, these glial cells are able to produce and release the endogenous ligands 2-AG and AEA and also express the intracellular degradation enzymes FAAH and MAGL (Stella et al., 1997; Walter et al., 2002; Vázquez et al., 2015; Grabner et al., 2016). CB_1_R activation in astrocytes not only controls their metabolic functions and signaling but also regulates synaptic transmission, through the tripartite synapse (Gorzkiewicz and Szemraj, 2018). When the electrical impulse causes neurotransmitter release from a presynaptic neuron, depolarization of the postsynaptic neuron occurs, leading to eCB release into the synaptic cleft and their binding to receptors located both in neurons and astrocytes (Stempel et al., 2016). While eCB binding to CB_1_R inhibits neurotransmitter release in presynaptic neurons, a process known as retrograde signaling (Stempel et al., 2016), it increases intracellular Ca^2+^ levels in neighboring astrocytes (Navarrete and Araque, 2010; Covelo and Araque, 2016). This Ca^2+^ increase stimulates glutamate release from astrocytes, which in turn causes a synaptic potentiation through mGluR1 receptors located in the presynaptic neuron. As the intracellular Ca^2+^ signal extends within astrocytes, it stimulates glutamate release in distal astrocyte regions, modulating in this way the synaptic transmission of many lateral synapses to the original source of eCBs (Figure 2A) (Navarrete and Araque, 2010; Covelo and Araque, 2016). In addition, CB_1_R activation in astrocytes also contributes to the regulation of CBF and the energy supply to neurons by increasing the glucose oxidation rate and ketogenesis (Shivachar et al., 1996; Bermudez-Silva et al., 2010; Stella, 2010; Jimenez-Blasco et al., 2020). Notably, most perivascular astrocytes express CB_1_R, highlighting their importance for CBF and metabolism (Rodriguez et al., 2001). On the other hand, despite CB_2_R expression in astrocytes is limited under physiological conditions, data show a significant upregulation of this receptor and the endocannabinoid tone in general upon different insults. Moreover, it also changes in neuroinflammatory conditions, suggesting an important role of the astroglial ECS in processes associated with brain damage and/or recovery (Shohami et al., 2011; Fernández-Ruiz et al., 2015; Cassano et al., 2017). In this sense, the study of different CBs has attributed them anti-oxidant and anti-inflammatory effects in experimental models of several pathologies (Cristino et al., 2020). In astrocytes, CBs regulate astrocyte activation and astrocyte-mediated neurotoxicity by reducing the release of inflammatory mediators and increasing prosurvival factors (Fernández-Ruiz et al., 2015; Estrada and Contreras, 2020). In different experimental settings, ECS modulation in astrocytes reduces TNF-α and IL-1β levels, which are upregulated following various inflammatory challenges (Grabner et al., 2016; Labra et al., 2018; Rodríguez-Cueto et al., 2018; Jia et al., 2020), suggesting that modulation of these cells with CBs could be contributing to neuroprotection through non-cell-autonomous mechanisms.

### Astrocyte ECS Pharmacological Modulation in Stroke

After an ischemic event, astrocytes are more resilient than neurons, being important for the post-acute phase because they preserve their viability and remain metabolically active both at the infarct core and penumbra regions (Thoren et al., 2005; Gürer et al., 2009). Considering the critical functions of these cells in the CNS, astrocytes are gaining notoriety as possible therapeutic targets for different neurological conditions including hypoxia and/or brain ischemia. At the same time, given their potent anti-oxidant and immunomodulatory effects, numerous studies have focused on the neuroprotective effects of CBs, mainly CBD, for stroke therapy (England et al., 2015). However, limited evidence is available concerning the mechanisms by which CBs modulate astrocyte function and astrocyte-mediated effects in the context of ischemic stroke. Here, we summarize the findings regarding the effects of CBs on astrocyte activation in the tMCAO, pMCAO and related models in adult animals, the HI model in neonate animals, and the oxygen and glucose deprivation/re-oxygenation (OGD/R) *in vitro* model of stroke.

In adult animals subjected to transient or permanent ischemia, the most consistent outcome in astroglial cells is increased astrogliosis, i.e., high GFAP immunoreactivity in CNS areas such as the motor cortex, the striatum, the hippocampus, or the spinal cord. Astrocytes with longer and wider projections, and other parameters that suggest a functional impairment of these cells are also observed (Figure 2B) (Hayakawa et al., 2008, 2009; Schiavon et al., 2014; Caltana et al., 2015; Kossatz et al., 2016; Ceprián et al., 2017; Jing et al., 2020). The inhibition of stroke-induced reactive gliosis was also observed in the pMCAO mouse model at 7 and 28 days after administering 1 mg/kg of ACEA (Caltana et al., 2015). In that study, CB_1_R expression was downregulated in ischemic conditions, which could be contributing to increase inflammation, neuronal degeneration, and astroglial reactivity, suggesting that upregulation of the eCB tone with ACEA could help revert these deleterious effects (Caltana et al., 2015). On the other hand, GFAP staining was significantly elevated in different brain areas of both adult wild type and CB_2_R^−/−^ mice after HI (Kossatz et al., 2016), and in rats subjected to HI in the spinal cord (Jing et al., 2020). In the study by Jing et al. (2020), i.p. pretreatment of rats with 1 mg/kg of the CB_2_R selective agonist JWH-133 1 h before ischemia not only inhibited astrocyte reactivity, determined by GFAP immunostaining but also reduced perivascular expression of TLR4/MMP9. Notably, TLR4 upregulation in astrocytes has been associated with a pro-inflammatory reactivity phenotype in astrocytes and with BBB disruption in the cortical devascularization brain ischemia model (Rosciszewski et al., 2018). The TLR4/MMP9-mediated reduction of astrocyte reactivity after ECS activation via CB_2_R is of special interest for stroke, as it has been suggested elsewhere that attenuation of the inflammatory process could be neuroprotective after tMCAO in rats (Piao et al., 2003). Although it remains to be determined whether the limitation of inflammation in those experimental conditions is mediated by astrocytes. In summary, in addition to having neuroprotective effects, the administration of different CB compounds like CBD, ACEA, and JWH-133 at various doses, duration of administration, and delivery methods prevented the increase in GFAP immunoreactivity, limiting astrocyte activation (Hayakawa et al., 2008, 2009; Schiavon et al., 2014; Caltana et al., 2015; Ceprián et al., 2017). In the majority of the aforementioned studies, the reduction of astroglial activation was observed with the administration of CBs that act through different receptors. For instance, while ACEA and JWH-133 are CB_1_R and CB_2_R agonists, respectively, it has been demonstrated that CBD preferentially binds to other receptors. Nevertheless, the precise molecular mechanism(s) by which the activation of the ECS is able to limit astrogliosis after stroke is not known yet and remains to be addressed experimentally. Moreover, there is scarce direct evidence showing that the modulation of astrocyte activity with CBs increases neuronal survival after stroke. Even so, a recent study in mice has shown that the increase in the eCB tone through the inhibition of FAAH and MAGL with the compound JZL195 (20 mg/kg, i.p.), induces long-term depression (LTD) at CA3-CA1 synapses in the hippocampus, and confers astrocyte-mediated neuroprotection after stroke (Wang et al., 2019). In that study, JZL195-induced LTD was used as a preconditioning insult to determine its potential neuroprotective effect against subsequent ischemia. Noteworthy, it was observed that preconditioning before tMCAO increased the number of surviving neurons through a mechanism dependent on a sequential activation of astroglial CB_1_R, and not neuronal CB_1_R, and postsynaptic glutamate receptors (Wang et al., 2019).

Over the years, the neuroprotective effects of CBD have been clearly demonstrated in experimental models of HI in rodents and notably in newborn pigs (Kicman and Toczek, 2020), but only a limited number of studies have characterized/evaluated astroglial reactivity as a neurological outcome. Evidence of GFAP immunoreactivity reduction after CBD treatment (5 mg/kg) has been reported both in newborn rats after tMCAO (Ceprián et al., 2017), and in newborn mice (1 mg/kg) after HI (Mohammed et al., 2017). In addition to decreasing perilesional gliosis volume in rats, CBD treatment limited astrocyte dysfunction, evidenced by the recovery of the *ex vivo* H^+^-MRS myoinositol/creatinine ratio, which had diminished after tMCAO (Ceprián et al., 2017). However, another work found that the number of activated astrocytes and IL-1β expression levels were downregulated in TRPV1^−/−^ neonatal mice following HI, indicating that TRPV1 is modulating astrocyte reactivity. These results might suggest that *in vivo*, the neuroprotective effects of CBD may not involve TRPV1 binding, at least in astrocytes (Yang et al., 2019)**.** On the other hand, in newborn pigs, some data indicate that CBD modulates astrogliosis after HI-induced brain damage. In the short term, i.v. administration of CBD (1 mg/kg) after acute HI promotes an increase in the number of astrocytes in the peri-infarct area (Pazos et al., 2013). However, by using the same animal model but conducting histological analyses 72 h after the induction of HI, CBD treatment (0.1 mg/kg) preserved the number, size, and morphology of GFAP-positive astrocytes in newborn pigs (Lafuente et al., 2011). Apart from analyzing GFAP reactivity by immunohistochemistry, some studies have detected high levels of the protein S100β, a possible biomarker of astrocyte damage, in the cerebrospinal fluid (CSF) of piglets after HI (Lafuente et al., 2011; Garberg et al., 2016, 2017). Noteworthy, in only one of those studies CBD administration decreased S100β levels (Lafuente et al., 2011), but this result might be explained by the lack of neuroprotective effects of i.v. administration of 1 mg/kg and 50 mg/kg of CBD observed in the works by Garber and co-authors (Garberg et al., 2016, 2017). Future research will clarify the ability of CBD to revert or not the increase in S100β levels observed after HI.

The most widely used experimental paradigm to investigate *in vitro* the effects of ischemia is OGD/R. Given the relevance of the BBB and the neurovascular unit in this pathology, the status of the ECS, as well as the effects of CBs have been studied in BBB models. In normoxia, the ECS has a modulatory role in BBB permeability in co-cultures of endothelial cells and astrocytes. In specific, AEA (10 µM) and OEA (10 µM) decrease BBB permeability through CB_2_R, TRPV1, CGRP, and PPARα receptors (Hind et al., 2015). Besides, in astrocyte monocultures, CBD diminishes IL-6 and vascular cell adhesion molecule-1 and increases lactate dehydrogenase release (LDH) values when administered at high concentrations (10 µM) (Hind et al., 2016). In these same *in vitro* BBB models, the eCB-like molecules OEA, PEA, and virodhamine (all 10 µM), as well as CBD (100 nM and 10 µM) attenuated the increase in BBB permeability induced by OGD/R (Hind et al., 2015; Hind et al., 2016). In a similar way, it was recently demonstrated that cannabidivarin (CBDV) and cannabigerol (CBG), two phytocannabinoids, are protective against OGD/R in human endothelial cells, astrocytes, and pericytes, the different cells that form the BBB (Stone et al., 2021). In astrocyte monocultures, CBG (10 nM–3 µM) and CBDV (30 nM, 1 and 3 µM) diminished IL-6 levels after OGD/R. And 1 and 3 µM of CBG and 10 nM, 1 and 3 µM of CBDV, reduced OGD/R-induced levels of LDH release, but the mechanisms by which these two compounds provide protection need to be further investigated (Stone et al., 2021). Despite the evidence indicating an active role of the ECS in regulating astrocyte metabolism *in vitro*, there is a generalized lack of evidence regarding the newest findings on the role of astrocytes as neuroprotectors or neurotoxic in this and other *in vitro* models of stroke or in *ex vivo* experiments. Overall, the evidence available so far indicates that the modulation of astrocyte function/reactivity with CBs could be used as a possible therapeutic approach to limit/arrest neurotoxic processes or promote recovery mechanisms in ischemic stroke (Figure 2C).

## Oligodendrocytes

**FIGURE 3:**
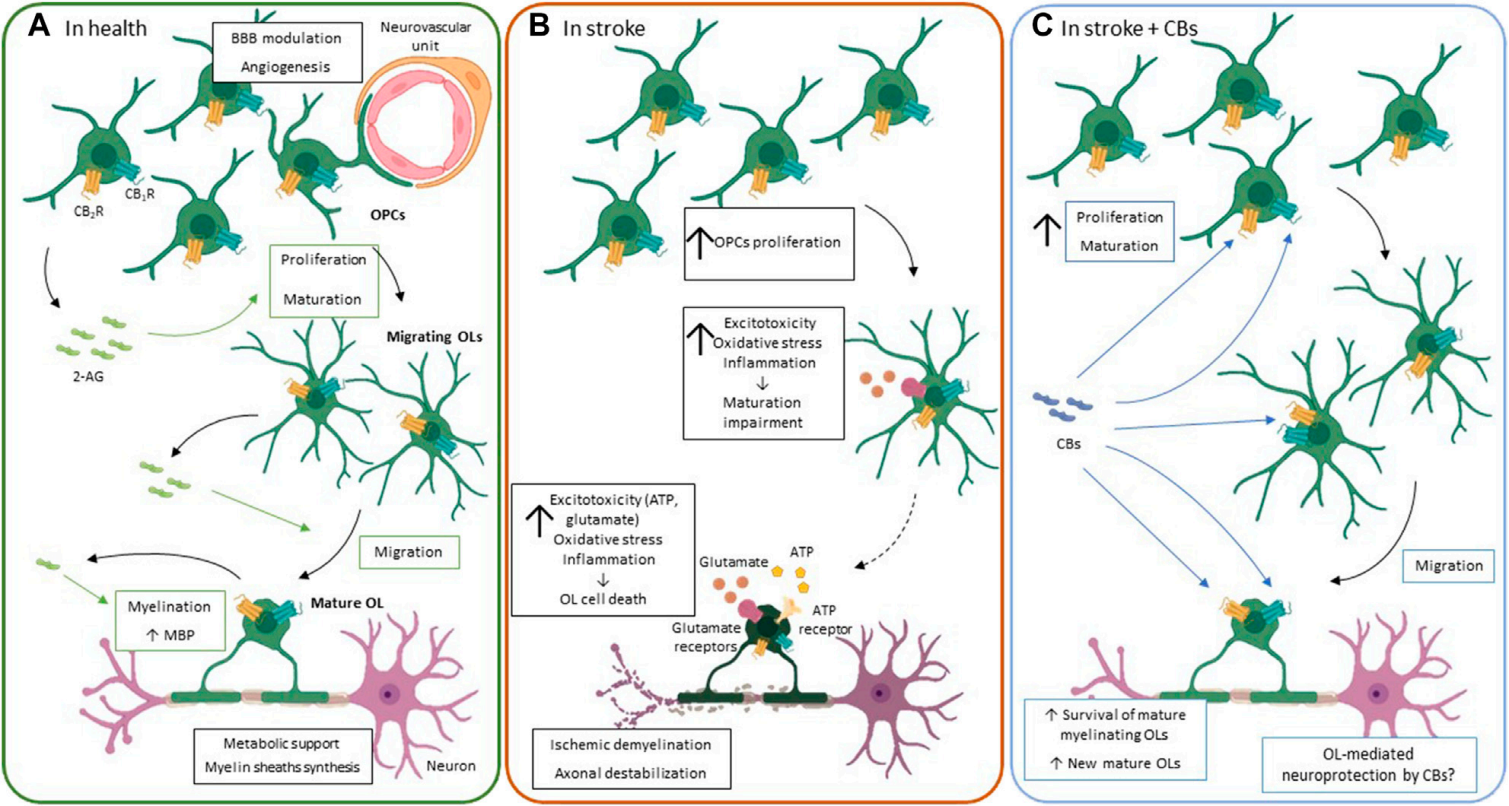
The ECS functioning in oligodendrocytes. **(A).** Under physiological conditions, oligodendrocytes play a key role in the metabolic support of axons, myelin sheath synthesis and BBB regulation, among others. In the CNS, the endocannabinoid system, particularly 2-AG, is involved in oligodendrocyte proliferation, maturation, migration and myelination of oligodendrocytes. 2-AG is produced in an autocrine manner and exerts its effects through its binding to CB_1_R and CB_2_R. **(B).** An ischemic event induces mature oligodendrocytes cell death due to the high sensitivity of these cells to: 1) glutamate and ATP receptor-induced excitotoxicity, 2) oxidative stress, i.e., high iron content and deficient antioxidant system, and 3) inflammation, through release of cytokines like TNF-α. In an attempt to repair the damage caused by stoke there is a strong oligoproliferative response. However, the maturation of these new oligodendrocytes is impaired, and they do not reach the mature myelinating oligodendrocyte stage, perpetuating myelinating deficits that contribute to motor and sensitive impairment observed after stroke. **(C).** Although the evidence of the oligoprotective potential of CBs after stroke is scarce, they seem to reduce the myelination impairment by 1) reducing oligodendrocyte cell death and 2) promoting oligodendrocyte proliferation and maturation into myelinating oligodendrocyte after the insult. Green arrows and boxes: eCB-mediated effects; blue arrows and boxes: CB-mediated effects. 2-AG: 2-Arachidonoylglycerol; BBB: blood-brain barrier; CB_1_R: cannabinoid receptor 1: CB_2_R: cannabinoid receptor 2; CBs: cannabinoids; MBP: myelin binding protein; OL: oligodendrocyte; OPC: oligodendrocyte progenitor cell.

Oligodendrocytes are the myelinating cells of the CNS. The myelin layers, composed mostly of water and lipids but also proteins, enwrap the axon and form a multilamellar compacted myelin sheath, protecting and isolating the axon (Morell and Quarles, RH, 1999). Myelin electrically isolates axons, allowing the saltatory impulse propagation and speeding the impulse transmission (Nave and Trapp, 2008). Besides this structural function, oligodendrocytes play a key role in the metabolic support of axons by producing lactate that is then transported to axons (Figure 3A) (Fünfschilling et al., 2012; Jha and Morrison, 2020). OPCs are widely distributed throughout the adult rat brain and participate in the modulation of the BBB and in angiogenesis (Dawson et al., 2003; Maki et al., 2015; Maki, 2017). Thus, oligodendrocytes are vital for brain circuit activity and neuron support, and their death and later remyelination failure have deleterious consequences in stroke outcome.

### Oligodendrocytes in Ischemic Stroke

Although the majority of studies on stroke focus on gray matter damage, the relevance of white matter injury has rapidly grown over the last years. Noteworthy, white matter injury occupies approximately half of the infarct area after a stroke (Ho et al., 2005), and myelinating disturbances resulting from stroke directly correlate with a poorer cognitive and motor outcome (Wang et al., 2016).

Oligodendrocytes are particularly susceptible to stroke due to their sensitivity to excitotoxicity and oxidative stress. In these cells, the expression of AMPA and NMDA receptors is developmentally regulated and correlates with their maturation from OPCs to mature myelinating oligodendrocytes (Káradóttir et al., 2005; Salter and Fern, 2005; Spitzer et al., 2019). The activation of AMPA and NMDA receptors in oligodendrocytes induces the retraction of their processes and causes oligodendrocyte cell death in the OGD model, effects that are prevented by blocking both receptors (Salter and Fern, 2005). Oligodendrocytes are also sensitive to the increase in the excitatory neurotransmitter ATP that takes place in stroke, through the P2X7 receptor (Domercq et al., 2009).

Oligodendrocytes are the brain cells with the highest concentration of iron, which is used to synthesize myelin (Reinert et al., 2019). This makes them extremely sensitive to variations in oxidative stress, as nicely reviewed elsewhere (Bresgen and Eckl, 2015). Furthermore, mature oligodendrocytes and, especially OPCs, are characterized by having limited antioxidant defenses (Fragoso et al., 2004; Spaas et al., 2021). This vulnerability is particularly strong in earlier stages of oligodendrocyte maturation, which may affect the stroke-induced oligoreparative response (Fragoso et al., 2004). Confirming this high oligodendrocyte sensitivity to stroke, oligodendrocyte cell death can be identified *in vivo* as early as 30 min after stroke (Pantoni et al., 1996). Meanwhile, alterations in the morphology of oligodendrocytes that survive have been described 24 h after insult (Mages et al., 2019). On the other hand, stroke induces a strong proliferative response of OPCs, which migrate to the affected area and mature into myelinating oligodendrocytes (Figure 3B) (Zhang et al., 2011; Bonfanti et al., 2017). This proliferative response seems to be age-dependent (Dingman et al., 2018). However, the proportion of the newly formed oligodendrocytes that reach a mature stage after stroke is surprisingly low (Bonfanti et al., 2017; Dingman et al., 2018). The mechanisms of this developmental impairment are not yet clear, although excitotoxicity, inflammation, and oxidative stress seem to play a key role (Figure 3B).

### Oligodendrocytes and the ECS

The ECS modulates oligodendrocyte maturation at every step: from the proliferation of OPCs, to their migration and maturation until the final step of myelination (Gomez et al., 2010; Fernández-López et al., 2012; Sanchez-Rodriguez et al., 2018; Tomas-Roig et al., 2020). In these cells, CB receptors are found along white matter tracts, and the first experiments activating CB_1_R showed that it promotes myelin basic protein (MBP) expression in the rat subcortical white matter (Herkenham et al., 1991; Arévalo-Martín et al., 2007). Particularly important for oligodendrocyte development is the constitutive production of 2-AG (Gomez et al., 2010, 2015; Sanchez-Rodriguez et al., 2018). The expression of the 2-AG synthesis enzymes, DAGL⍺ and DAGLβ, is higher in OPCs than in mature oligodendrocytes, whereas the degradation enzyme MAGL is upregulated in mature oligodendrocytes The effect of 2-AG in these cells is mediated by CB_1_R/CB_2_R (Figure 3A) (Gomez et al., 2010). The administration of different antagonists of these receptors reduces oligodendrocyte proliferation and migration. It also impairs oligodendrocyte maturation, revealed by a reduced arborization of immature oligodendrocytes, and myelin production *in vitro*, with lower expression levels of MBP and myelin-associated glycoprotein (Gomez et al., 2011, 2015; Sanchez-Rodriguez et al., 2018). Actually, CB_1_R^−/−^ animals are characterized by having less cell proliferation, evidenced by BrdU^+^ cells, in the subventricular zone (SVZ) and in the dentate gyrus of rats (Jin et al., 2004). Although the molecular pathways associated with these effects are not well characterized, the PI3K/mTOR pathway has been proved to be involved in the proliferative effect of 2-AG in oligodendrocytes, and the ERK/MAPK signaling pathway has been associated with oligodendrocyte maturation (Gomez et al., 2010, 2011).

Interestingly, the pharmacological modulation of the ECS has direct effects on oligodendrocyte maturation and migration, and increased survival of OPCs has been observed in models of white matter injury. The *in vitro* administration of the MAGL inhibitor JZL-184 at 1 mg/kg, which increases 2-AG levels, accelerates oligodendrocyte differentiation, and increases the percentage of migrating cells (Gomez et al., 2010; Sanchez-Rodriguez et al., 2018). Indeed, it has been observed that the direct administration of 2-AG promotes oligodendrocyte migration (Sanchez-Rodriguez et al., 2018). The therapeutic effects of 2-AG have also been described in pathologies like spinal cord injury. For instance, the administration of 5 mg/kg of 2-AG 30 min after a moderate contusive SCI in rats reduced white matter injury and promoted oligodendrocyte survival, even 28 days after injury (Arevalo-Martin, Garcia-Ovejero and Molina-Holgado, 2010). Furthermore, the inhibition of 2-AG degradation with the MAGL inhibitor UCM03025 (5 mg/kg) improved motor impairment and recovered MBP expression in a multiple sclerosis model, with higher BrdU^+^/Olig2^+^ cells, i.e., new OPCs, in the spinal cord of affected mice that were treated with the compound (Feliú et al., 2017).

CB_1_R/CB_2_R selective agonists or even non-selective agonists, such as WIN55,212-2, also promote OPCs proliferation, oligodendrocyte maturation, with cells showing a more complex morphology, and myelinization, by increasing MBP production (Arévalo-Martín et al., 2007; Gomez et al., 2011, 2015; Tomas-Roig et al., 2020). The daily administration of 0.5 mg/kg of WIN55,212-2 prevented demyelination and promoted remyelination, increasing the number of myelinated axons, in a cuprizone model of demyelination in mice (Tomas-Roig et al., 2016; Tomas-Roig et al., 2020). However, the CB dose should be thoroughly tested, as the daily administration of 1 mg/kg potentiated axonal demyelination, probably due to a downregulation of CB_1_R (Tomas-Roig et al., 2016; Tomas-Roig et al., 2020).

CB_2_R activation has also been shown to be oligoprotective *in vitro* with the CB_2_R agonist BCP. This compound reduced LPS-induced oligodendrocyte death by decreasing oxidative stress and TNF-α (Askari and Shafiee-Nick, 2019). Actually, administration of tetrahydrocannabinol (THC), the main psychoactive compound in *Cannabis sativa*, for 5 days at 3 mg/kg in the cuprizone mouse model, reduced myelin loss and improved motor impairment (Aguado et al., 2021). In this study, electron microscopy analysis showed lower g-ratios in the THC-treated group versus control, indicating that THC effect is on remyelination (Aguado et al., 2021). CBs can also promote oligodendrocyte survival by CB_1_R and CB_2_R independent mechanisms. For example, 1 μM CBD was able to prevent oligodendrocyte death induced by inflammatory and oxidative stress stimuli through the reduction of endoplasmic reticulum stress in primary cell cultures (Mecha et al., 2012).

In summary, the evidence suggests that, due to its role in promoting oligodendrocyte lineage survival and remyelination, the ECS is a promising therapeutic target for functional recovery after demyelinating pathologies, including stroke.

### Oligodendrocyte ECS Pharmacological Modulation in Stroke

Despite the aforementioned data on the possible therapeutic effects of the ECS modulation in oligodendrocytes, very few works have explored either the ECS system itself or the oligoprotective potential of CBs during or after an ischemic event. In agreement with the above-mentioned results, the administration of WIN55,212-2, at the high concentration of 9 mg/kg, increased the proliferation rate of OPCs in the ipsilateral SVZ of adult rats 24 h after pMCAO (Sun and Fang, 2013). Moreover, WIN55,212-2 was also able to increase the number of NG2^+^-OPCs within the stroke penumbra and reduce the NG2^+^/caspase-3^+^ cells during 14 days post-damage in a pMCAO model, an effect that could be related to the increased expression of CB_1_R in that area (Sun and Fang, 2013). Interestingly, this increased proliferation/protection in OPCs seemed to translate into new mature myelinating oligodendrocytes, an effect that was partially mediated by CB_1_R (Figure 3C). In addition, the amelioration of MBP loss was also prevented via CB_1_R activation and that was associated with an increase in the number of myelinated axons and lower g-ratio values (Sun and Fang, 2013; Sun and Fang, 2013). In a rodent model of neonatal HI, 1 mg/kg WIN55,212-2 also promoted oligodendrocyte proliferation in the SVZ up to 14 days after the insult. This increase positively correlated with the presence of new APC^+^/BrdU^+^ mature oligodendrocytes in the injured dorsal striatum observed 28 days after the damage. In addition, an upregulation of CB_2_R expression was observed in the SVZ in the short term; however, it is not known whether WIN55,212-2 modulates the expression of this receptor in the SVZ (Fernández-López et al., 2010). This CB-mediated protection of oligodendrocytes and myelin has also been observed with the administration of CBD, a compound with excellent antioxidant and anti-inflammatory properties (Atalay et al., 2019). Notably, the administration of the low dose of 1 mg/kg CBD in a neonatal model of HI was oligoprotective in the ipsilateral cortex and corpus callosum. Similar to what happens in humans, the hypomyelination induced by the insult was directly related to the motor and cognitive impairment outcomes. Interestingly, CBD treatment reduced insult-induced oligodendrocyte impairment and preserved myelin (Figure 3C) (Ceprián et al., 2019).

We can conclude from these studies that the modulation of the oligodendroglial ECS is a promising field for the treatment of myelin disturbances associated with stroke and its motor/cognitive sequelae. Although more experimental evidence is necessary, the results obtained so far in stroke and other pathologies that share key points in pathophysiology, have shown that CBs may help reduce myelin loss, promote oligodendrocyte development and/or recovery, and eventually reduce the behavioral impairment associated with stroke.

## Concluding Remarks

In recent years, significant efforts have been devoted to searching for new therapeutic options aimed at limiting/arresting the post-stroke inflammatory response, including the possibility of modulating glial cells. These cells, mainly microglia and astrocytes, are gaining notoriety as potential therapeutic targets in part because they exert critical functions in the CNS and rapidly respond to the lack of blood supply to the brain, contributing to the subsequent immune response. In this review, we have summarized the role and function of microglia, astrocytes, and oligodendrocytes in homeostatic conditions and their response to harmful challenges like an ischemic event.

The ECS, among other physiological functions, participates in the regulation of the immune system and the inflammatory response in the healthy brain, and substantial evidence suggests a neuroprotective effect after the pharmacological manipulation of this system in stroke. However, the experimental evidence regarding the possible therapeutic effects of manipulating the ECS in glial cells is promising but less abundant. Thus, we have also analyzed the evidence of pharmacologically modulating the ECS on microglia, astrocytes, and oligodendrocytes in the context of ischemic stroke.

As we have reviewed here, glial cells are highly dynamic and complex heterogeneous cells that work synergistically and in a highly coordinated way in the CNS. Their role in the pathophysiology of stroke is extraordinarily complex and depends on the timing after stroke, the affected region, the inherent heterogeneity of glial cells, and the interactions between different cell types. Therefore, we must take into account that similar to what occurs in other pathological conditions, an ischemic event perturbs their functioning both individually and collectively. In this sense, it is fundamental to comprehend and consider that in addition to inducing changes that are region-specific, hypoxia alters neuron-glia intercommunications at all levels. Thus, new approaches targeting glial cells in stroke should also investigate the interplay among all the aforementioned cellular players; microglia-astrocytes-oligodendrocytes-neurons. These future studies should also consider evaluating intercellular interactions at all possible levels, morphological, functional, and even metabolically.

The ECS seems to have a significant role in the modulation of glial cell function, not only on cell reactivity or phenotype polarization but also by promoting cell survival and/or preventing their functional impairment. Notably, all glial cells described in this review express to a greater or lesser degree the different elements that integrate the ECS. Indeed, ischemic stroke induces an imbalance of different elements of the glial ECS that would potentially have an impact on neurons and other glial cells, most probably altering cell-to-cell interactions as well. Recently, microglia-astrocyte crosstalk has gained attention, due to the putative dual effect that these cells have under pathological conditions, being able to incline the balance toward a neuroprotective or a neurodegenerative environment. Thus, it is likely that in addition to having beneficial effects on each cell type individually, the pharmacological manipulation of the ECS would have a positive impact on intercellular interactions that would in turn contribute to inclining the balance toward a neuroprotective microenvironment. However, due to the dual effects reported in astrocytes/microglia in stroke, the precise molecular mechanisms underlying the effects of the ECS modulation in these cells and their interaction with other cells must be thoroughly investigated. Further studies are needed to understand the precise role of the ECS on glial cell function and to consider if CBs could be used as therapeutic agents aimed at protecting glial cells per se or if CB-treated glial cells could be used as a neurorepair strategy for stroke.

Finally, we are now beginning to understand the significance of white matter damage in stroke, the sensitivity of oligodendrocytes to the insult, and the impaired remyelination directly related to stroke’s sequelae. In that sense, the ECS and its modulation seem to be a promising target to promote oligodendrocyte survival and remyelination, not only because it directly influences oligodendrocyte maturation and myelination; but also by an indirect influence on astrocytes and microglia, promoting an improvement of the microenvironment that in turn could induce a successful oligodendrocyte remyelinating response.

In summary, due to the profound implication of glial cells in stroke, the pharmacological modulation of the glial ECS could represent a significant advantage to help reduce/limit neuronal damage and stroke-associated sequelae.

## Abbreviations

AEA, N-arachidonoylethanolamine or anandamide; BBB, blood-brain barrier; BCP, β-caryophyllene; CBD, cannabidiol; CBDV, cannabidivarin; CBG, cannabigerol; CB, cannabinoid; CB_1_R, cannabinoid receptor type 1; CB_2_R, cannabinoid receptor type 2; CBF, cerebral blood flow; CNS, central nervous system; DAGL, diacylglycerol-lipase; eCB, endocannabinoid; ECS, endocannabinoid system; GFAP, glial fibrillary acidic protein; HI, hypoxia-ischemia; IL-1β, interleukin-1beta; intraperitoneal, i.p.; intravenous, i.v.; LPS, lipopolysaccharide.
